# Supplementary material for: Memory B cells are reactivated in subcapsular proliferative foci of lymph nodes
Source: Nat Commun. 2018 Aug 22;9:3372. doi: 10.1038/s41467-018-05772-7 (PMC6105623; doi:10.1038/s41467-018-05772-7)
Supplement: Supplementary file 1 — Supplementary Information [file 41467_2018_5772_MOESM1_ESM.pdf]

Supplementary Information

Memory B Cells Are Reactivated in Subcapsular Proliferative Foci of Lymph Nodes

Moran et al.

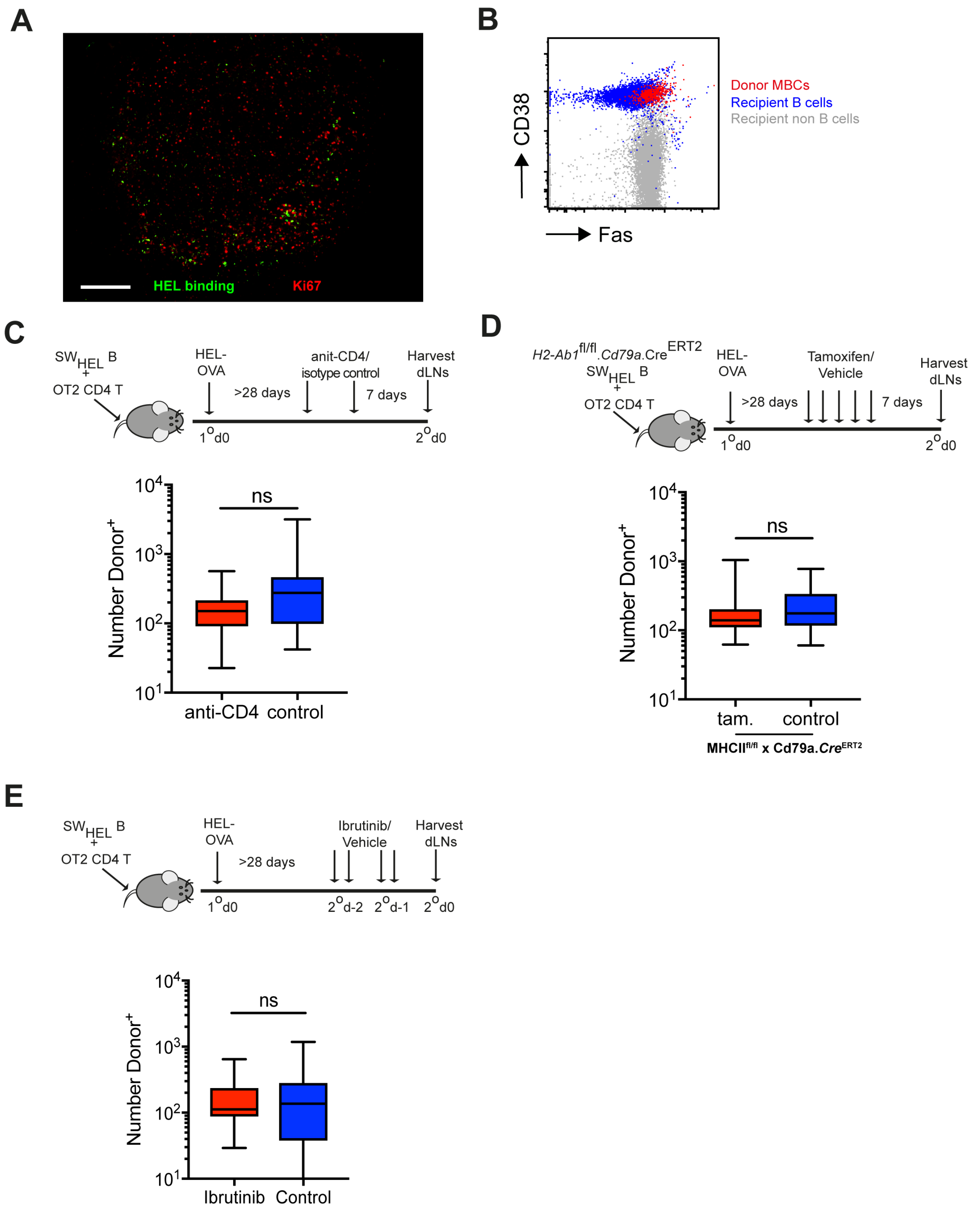

Supplementary Figure 1. Resolution of primary immune response in memory phase.

(A) Representative histological section of lymph node >28 days after primary immunization. HEL binding (green), Ki67 (red). Scale bar = 100μm.

(B) Expression of CD38 and Fas on donor-derived HEL^+ MBCs (red) compared to naïve endogenous recipient B cells (blue) and endogenous non-B cells (grey).

(C) Schema for T cell depletion with anti-CD4 mAb and enumeration of number of donor HEL^+ cells in memory response. Data is representative of 2 experiments with n=5 each group. Comparison between groups utilised one-tailed unpaired Student's test.

(D) Schema for inducible deletion of MHCII on MBCs and enumeration of number of donor HEL^+ cells in memory response. Data is representative of 2 experiments with n=5 each group. Comparison between groups utilised one-tailed unpaired Student's test.

(E) Schema for ibrutinib treatment and enumeration of number of donor HEL^+ cells in memory response following ibrutinib treatment. Data is representative of 2 experiments with n=5 each group. Comparison between groups utilised one-tailed unpaired Student's test. Box plots show centre line as median, box limits as upper and lower quartiles, whiskers as minimum to maximum values.

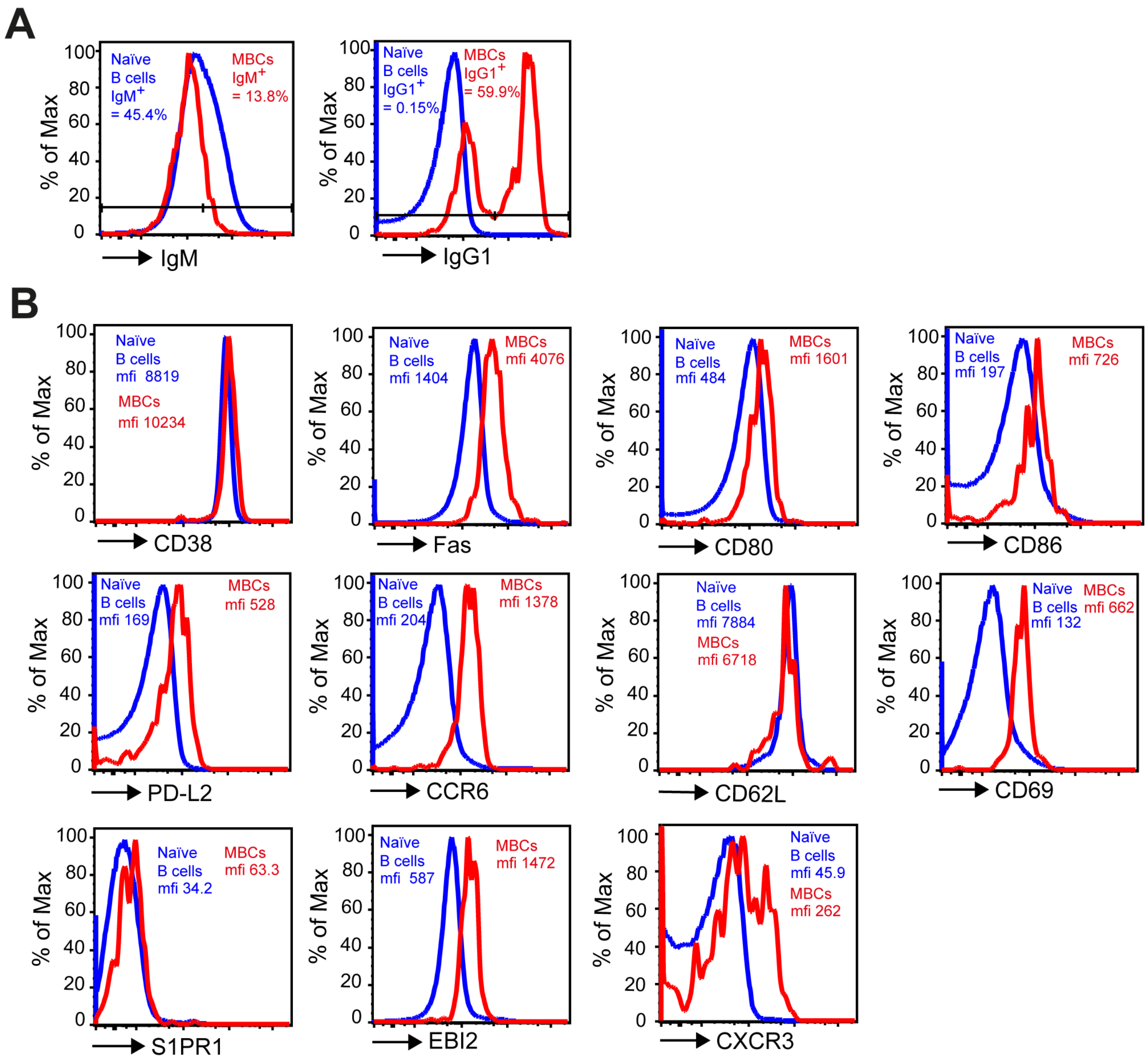

Supplementary Figure 2. MBC cell surface phenotype.

(A) Expression of immunoglobulin isotype on donor-derived HEL^+ MBCs (red) compared to naïve endogenous B cells (blue).

(B) Expression of cell surface markers on donor-derived HEL^+ donor-derived MBCs (red) compared to naïve endogenous B cells (blue). Data for MBCs are concatenated from 5-15 mice and naïve endogenous B cells are shown from one representative mouse. Data are representative of 2-5 independent experiments.

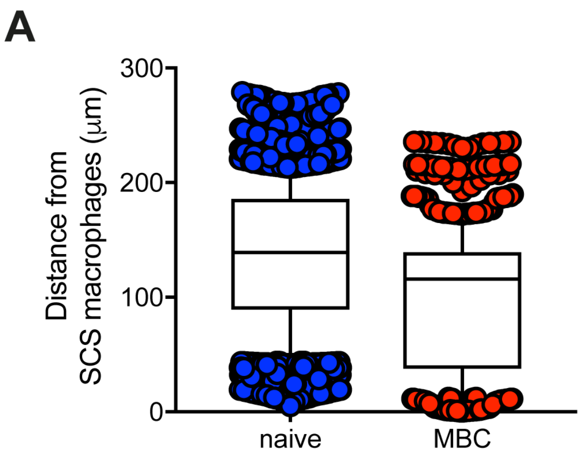

Supplementary Figure 3. Detection of cells deep in follicle using TPM.

(A) Distance of naïve and MBCs from the capsule surface as measured by the Immune Atlas plugin in Imaris. n=7116 naïve B cells and n=965 MBCs. Comparison between groups utilised one-tailed unpaired Student's test. Box plots show centre line as median, box limits as upper and lower quartiles, whiskers as minimum to maximum values.

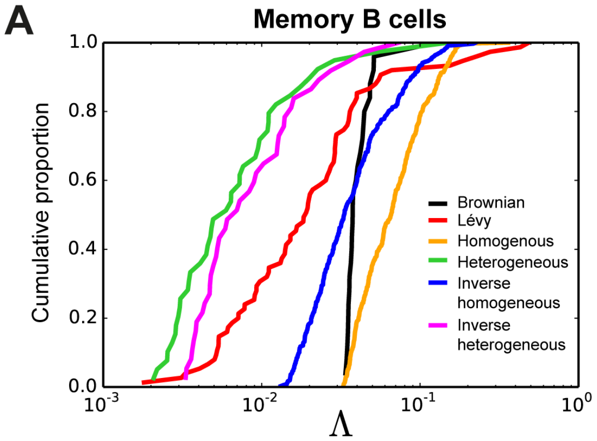

Kolmogorov-Smirnov differences

	Brownian	Levy	HomoCRW	HeteroCRW	invHomoCRW	invHeteroCRW
Brownian	-	0.60	0.41	0.89	0.42	0.91
Levy		-	0.62	0.45	0.33	0.41
HomoCRW			-	0.89	0.42	0.92
HeteroCRW				-	0.77	ns
invHomoCRW					-	0.70
invHeteroCRW						-

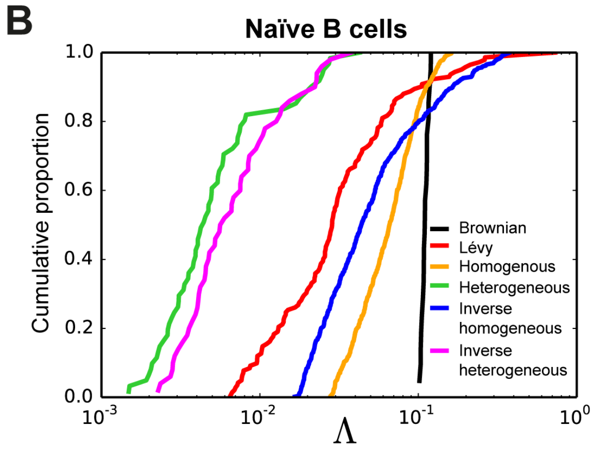

Kolmogorov-Smirnov differences

	Brownian	Levy	HomoCRW	HeteroCRW	invHomoCRW	invHeteroCRW
Brownian	-	0.90	0.85	1.00	0.75	1.00
Levy		-	0.58	0.70	0.35	0.60
HomoCRW			-	0.95	0.26	0.96
HeteroCRW				-	0.84	0.30
invHomoCRW					-	0.83
invHeteroCRW						-

Supplementary Figure 4. Modelling of naïve and MBC motility.

(A) and (B) Naïve and MBC population motilities are best modelled as a heterogeneous population performing a correlated random walk. Six random walk models were fitted against *in situ* data (see Methods for full details). Each was simulated in a 3D agent-based simulation, and using multi-objective optimisation we identified model parameters that best aligned *in situ* and *in silico* measures of cell speeds, turn speeds and mean squared displacements. Putative parameter values deliver trade-offs in their alignment against each metric. A set of parameter values' ability to deliver close and equal alignment across all metrics is quantified through Λ scores, with low values indicating superior capture. These are shown for each model as cumulative distribution plots, and statistically significant ($p<0.01$) Kolmogorov-Smirnov D values are shown. The motility of both B cell populations were most robustly captured as heterogeneous correlated random walks (CRW) that allow cells to differ in their inherent speeds and directionalities. That these models outperformed homogenous CRW highlights the importance of cellular heterogeneity in shaping the population's motility patterns. Inverse CRW explicitly dissuade walkers from simultaneously moving and turning quickly. Whilst inverse- outperformed standard-homogeneous CRW, this was not the case for the heterogeneous CRW formulations, suggesting this inverse relationship is not a critical feature in shaping the populations' motility patterns. Whilst a single set of parameter values

conferred Lévy walk competitive-with-best capture of memory B cell data, it was otherwise sub-optimal. We do not consider either B cell population to be performing a Lévy walk.

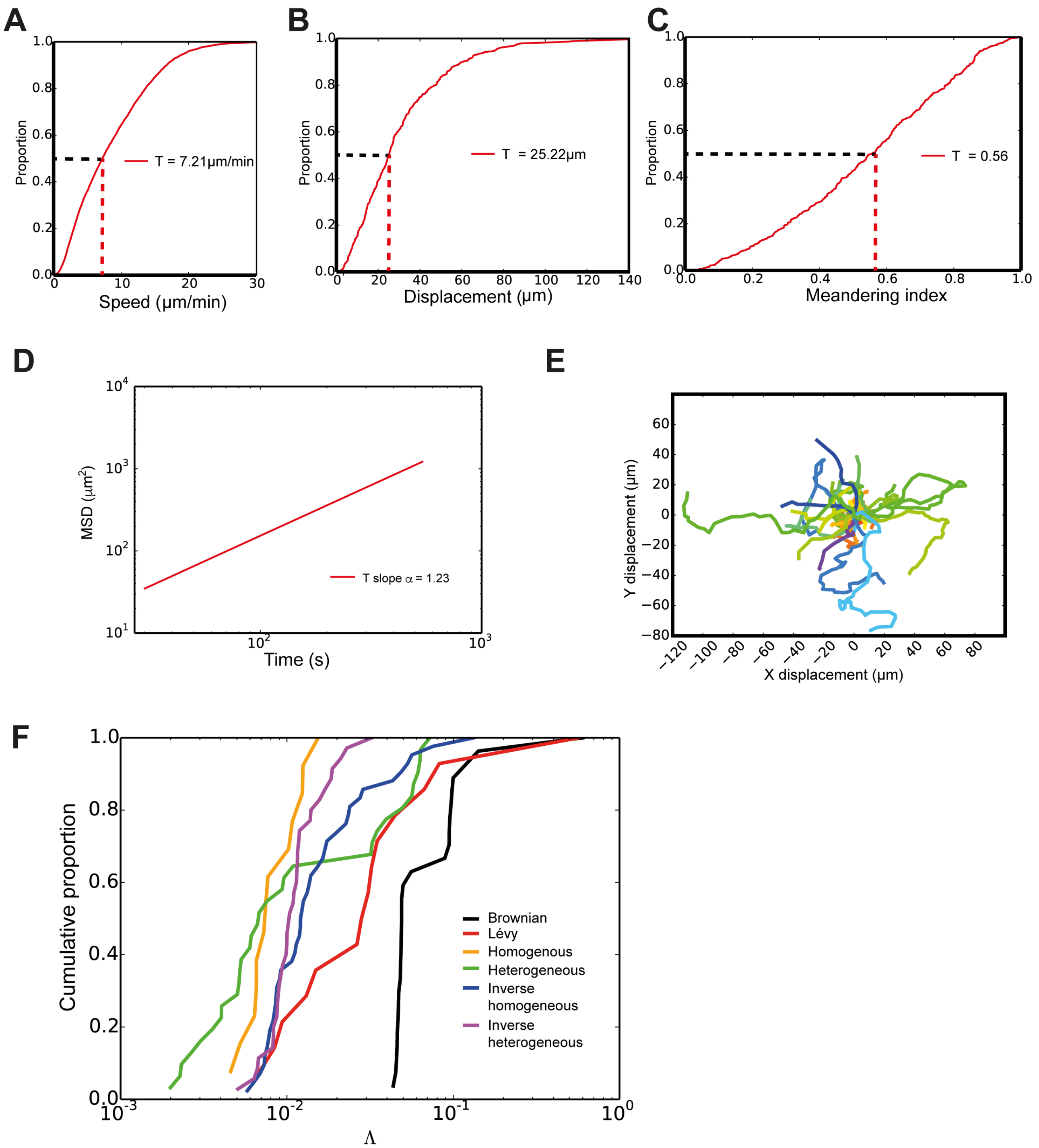

Kolmogorov-Smirnov differences

	Brownian	Lévy	HomoCRW	HeteroCRW	InvHomoCRW	InvHeteroCRW
Brownian	-	0.75	1	0.77	0.88	1
Lévy		-	0.71	ns	ns	0.61
HomoCRW			-	ns	ns	ns
HeteroCRW				-	0.47	0.43
InvHomoCRW					-	ns
InvHeteroCRW						-

Supplemental Figure 5. Secondary Tfh cells use a superdiffusive search strategy.

(A) Cumulative distribution function (CDF) plot of speed for secondary Tfh cells.

(B) CDF plot of displacement for secondary Tfh cells.

(C) CDF plot of meandering index for secondary Tfh cells.

(D) Mean squared displacement (MSD) plot of secondary Tfh cells. All data is pooled from three independent movies.

(E) Representative tracks for secondary Tfh cells. Data is representative of >3 independent experiments.

(F) Secondary Tfh cell populations' motilities are best captured through heterogeneous correlated random walks. Random walk motility models were fitted against these populations' motility data as described in Supplementary Figure 2.

A

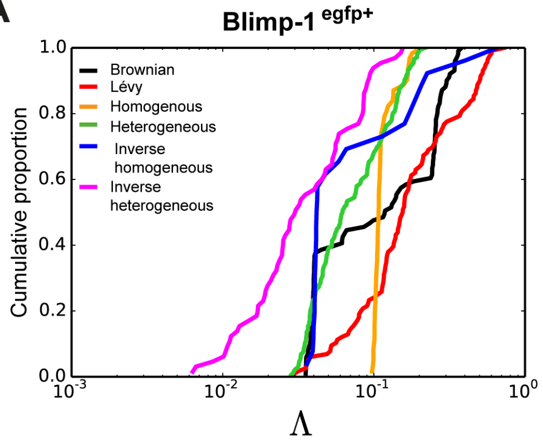

Kolmogorov-Smirnov differences

	Brownian	Levy	HomoCRW	HeteroCRW	invHomoCRW	invHeteroCRW
Brownian	-	0.94	0.52	1.00	0.61	1.00
Levy			0.68	0.60	0.46	0.85
HomoCRW			-	0.86	0.24	0.93
HeteroCRW				-	0.84	0.54
invHomoCRW					-	0.93
invHeteroCRW						-

B

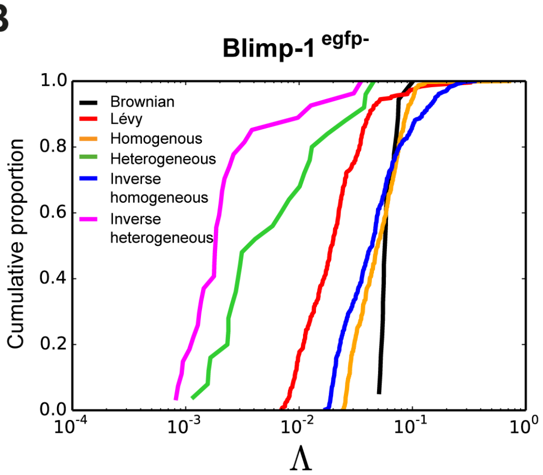

Kolmogorov-Smirnov differences

	Brownian	Levy	HomoCRW	HeteroCRW	invHomoCRW	invHeteroCRW
Brownian	-	0.93	0.47	0.41	ns	0.55
Levy		-	0.49	0.47	0.57	0.71
HomoCRW			-	0.66	0.69	0.92
HeteroCRW				-	ns	0.48
invHomoCRW					-	0.55
invHeteroCRW						-

Supplementary Figure 6. Modelling of Blimp-1^{+} and Blimp-1^{neg} cell motility in the SPF.

(A) SPF B cell populations' motilities are both best captured through heterogeneous correlated random walks. Random walk motility models were fitted against these populations' motility data as described in Supplementary Figure 2. Heterogeneous and inverse heterogeneous CRW together outperform all other models in capturing Blimp-1^{egfp+} population motility suggesting that this population's motility dynamics are critically shaped by a population of cells differing in their inherent directionalities and speeds. The inverse formulations of both the heterogeneous and homogeneous CRW outperform their standard counterparts, suggesting that the disinclination of these cells to simultaneously make fast movements and large reorientations is important in shaping the population's motility.

(B) Blimp-1^{egfp-} population motility is best captured as an inverse heterogeneous CRW, suggesting a population of cells heterogeneous in their inherent speed and directionalities that do not perform fast movements and large reorientations simultaneously. This population is poorly described by the Lévy walk.

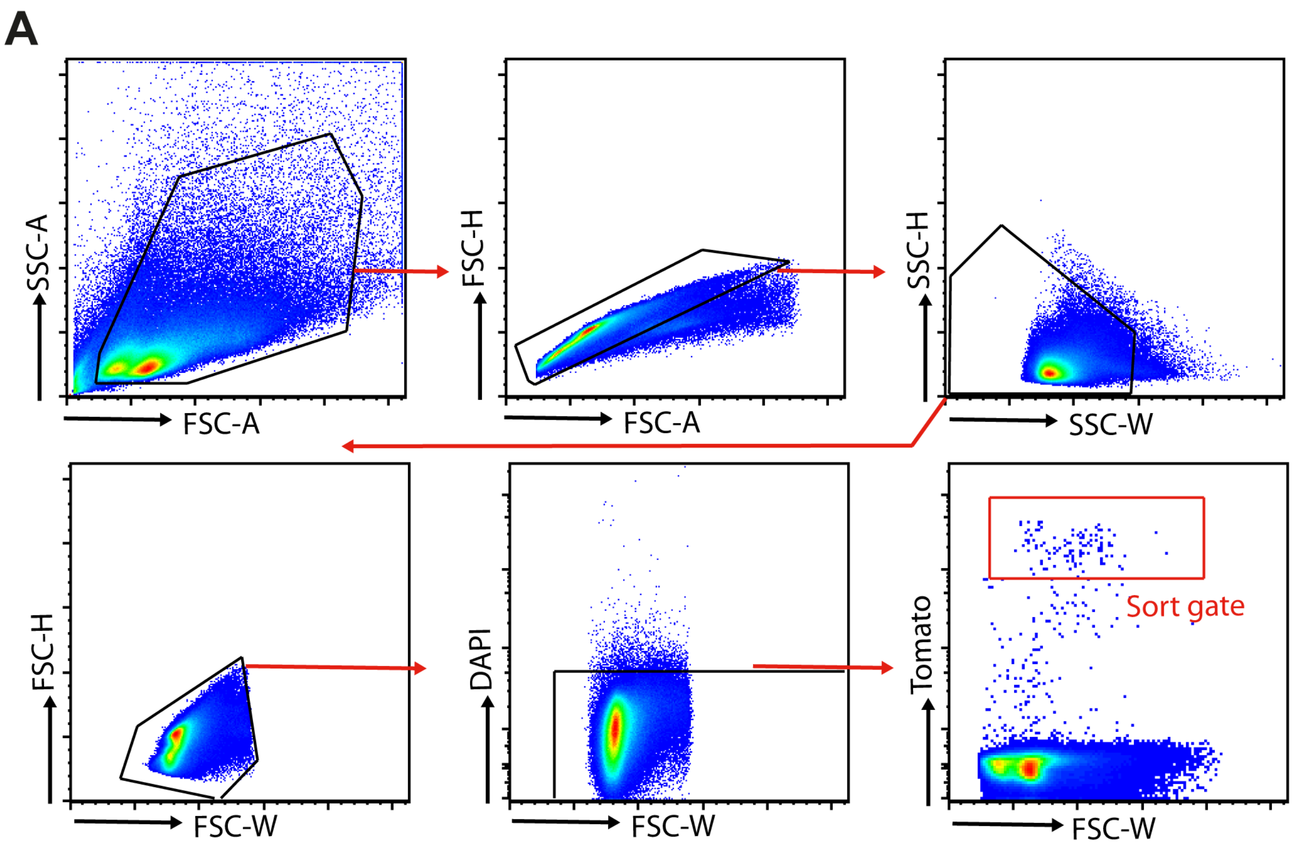

Supplementary Figure 7. Sorting strategy of donor-derived SW$_{HEL}$ B cells for scRNAseq.

(A) On day 5 post-recall, donor-derived Tomato SW$_{HEL}$ B cells were sorted for scRNAseq by gating for lymphocytes, excluding doublets and dead cells and sorting for tdTomato expressing donor cells.

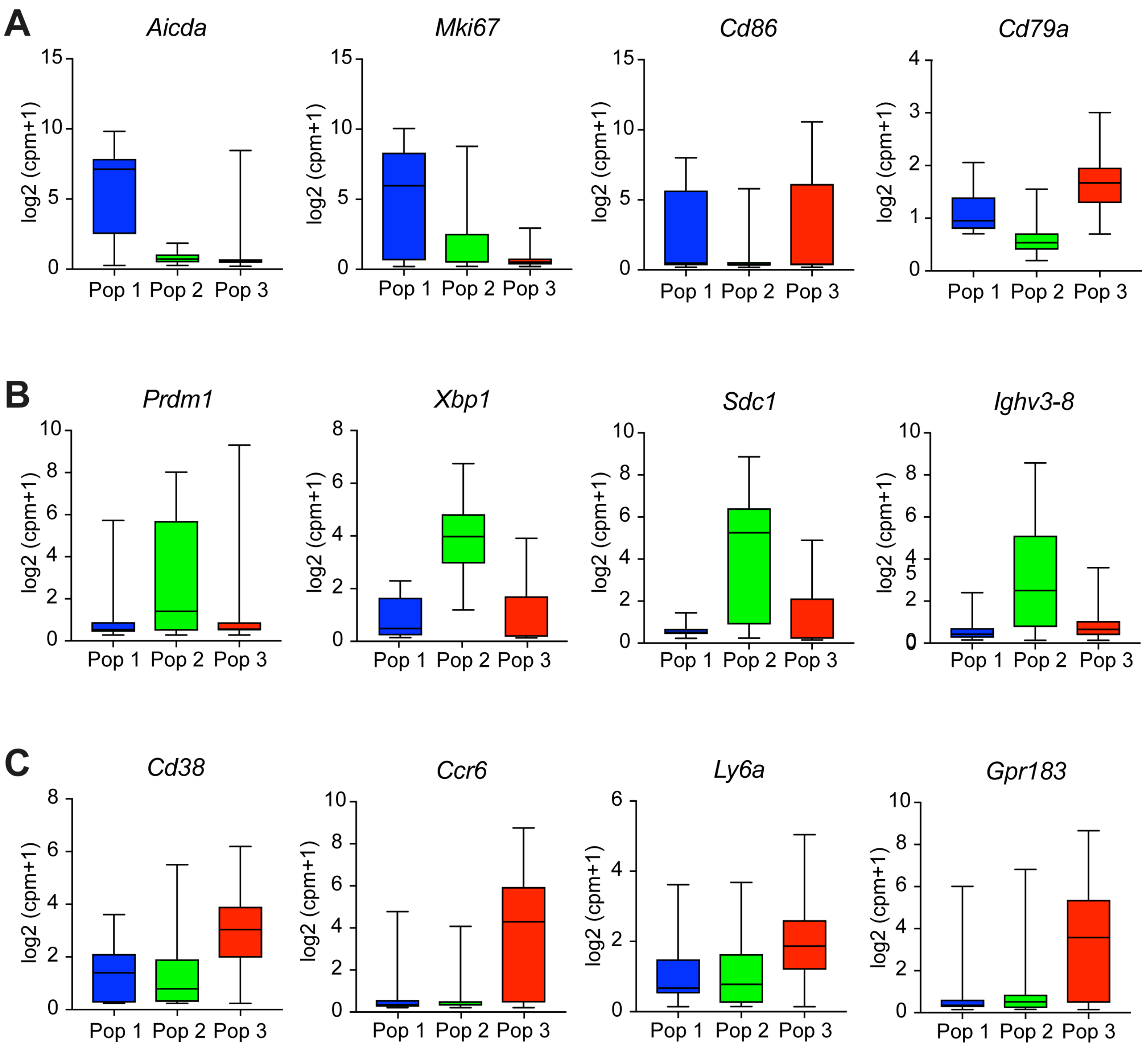

Supplementary Figure 8. Single cell expression of select genes by population 1, 2 and 3.

(A) Expression of select genes that contribute to metagene 1 that defines population 1. See also Supplementary Data File 1.

(B) Expression of select genes that contribute to metagene 2 that defines population 2. See also Supplementary Data File 2.

(C) Expression of select genes that contribute to metagene 3 that defines population 3. See also Supplementary Data File 3. Box plots show centre line as median, box limits as upper and lower quartiles, whiskers as minimum to maximum values.

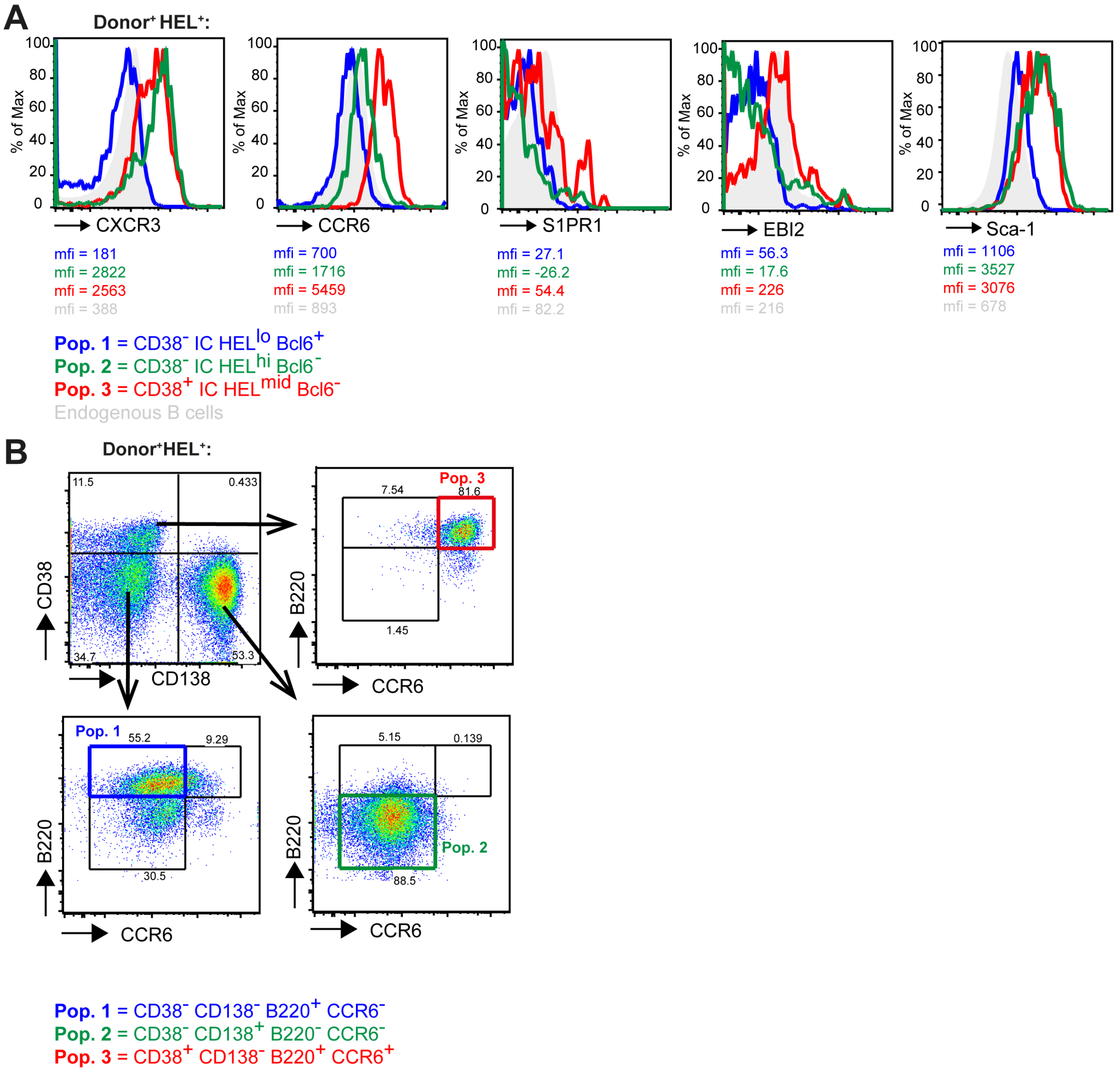

Supplementary Figure 9. Cell surface phenotype of SPF cells.

(A) Histogram of CXCR3, CCR6, S1PR1, EBI2 and Sca-1 expression on population 1 (CD38^{lo} IC HEL^{lo} Bcl6^{+}), population 2 (CD38^{hi} IC HEL^{mid} Bcl6^{-}), population 3 (CD38^{lo} IC HEL^{hi} Bcl6^{-}) and endogenous B cells, 5 days after HEL-OVA re-immunization.

(B) Alternative gating strategy using surface markers only to identify the same 3 populations in the secondary response, 5 days after HEL-OVA re-immunization.

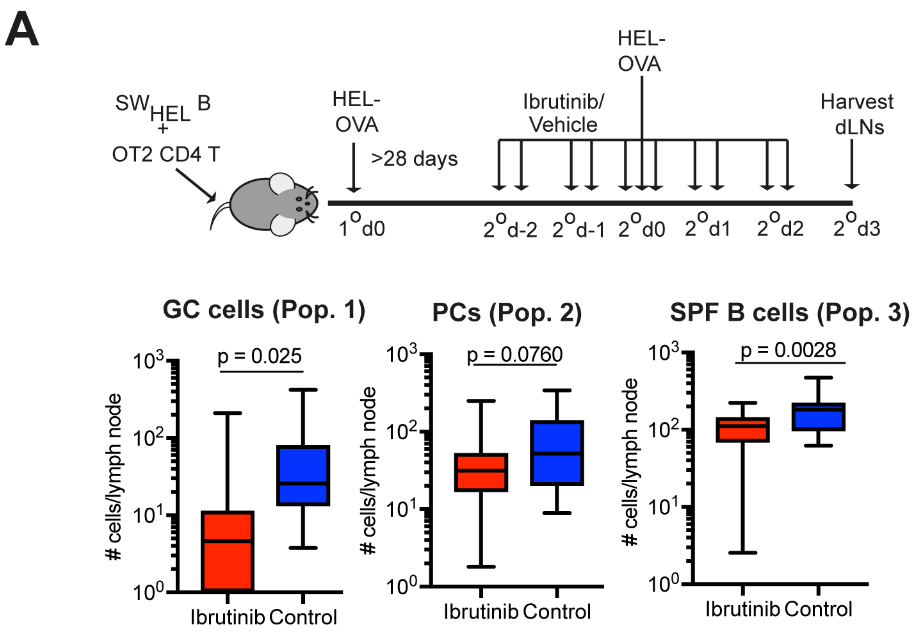

Supplementary Figure 10. Btk-mediated BCR signalling is required for reactivation of MBCs and generation of cells in the SPF.

(A) Schema for blocking of Btk-mediated BCR signaling before and during MBC reactivation and enumeration of population 1, 2 and 3 in the secondary response. Data is representative of 2 experiments with n=5 each group. Comparison between groups utilised one-tailed unpaired Student's test. Box plots show centre line as median, box limits as upper and lower quartiles, whiskers as minimum to maximum values.

Supplementary Table 1 – Antibodies and reagents

TARGET	CLONE	CONJUGATION	SOURCE	CATALOGUE NUMBER	DILUTION
**Mouse**					
B220	RA3-6B2	BV650	Biolegend	103241	1/300
		biotin, Pacific Blue, BV786	BD Biosciences	553086, 558108, 563894	1/400
Bcl6	K112-91	PE	BD Biosciences	561522	1/30
CCR6	29-2L17	PE-Cy7	Biolegend	129803	1/200
CD11b	M1/70	biotin	BD Biosciences	553309	1/200
CD11c	HL3	biotin	BD Biosciences	553800	1/200
CD16/32	2.4G2	purified	BioXCell	BE0307	1/400
CD138	281-2	PE, BV650	BD Biosciences	553714, 564068	1/300
CD169	SER-4	purified	UCSF Hybridoma Core	N/A	N/A
CD35	8C12	biotin	BD Biosciences	553816	1/200
CD38	90	BV510, FITC, PerCPCy5.5	BD Biosciences	740129, 558813, 562770	1/300
CD4	GK1.5	biotin	BD Biosciences	553728	1/200
CD43	S7	biotin	BD Biosciences	553269	1/200
CD45.1	A20	FITC , PE-Cy7	eBioscience	11-0453-82, 25-0453-82	1/300
		PerCPCY5.5	Biolegend	110728	1/300
CD45.2	104	BUV395	BD Biosciences	564616	1/400
CD62L	MEL-14	FITC	BD Biosciences	561917	1/200
CD69	H1.2F3	FITC	BD Biosciences	557392	1/200
CD8	53-6.7	biotin	BD Biosciences	553028	1/200
CD80	16-10A1	BV650	BD Biosciences	563687	1/300
CD86	GL-1	BV650	Biolegend	105035	1/300
CXCR3	CXCR3-173	BV421	Biolegend	126521	1/300
EBI2		biotin	Conjugated in house	N/A	N/A
Fas	Jo2	PE-Cy7, biotin	BD Biosciences	557653, 554256	1/400
HyHEL9		A647	Conjugated in house	N/A	1/400 – 1/800
IgG1	A85-1	biotin	BD Biosciences	553441	1/400
IgM	II-41	PE-Cy7	eBioscience	25-5790-81	1/400
Ki67	SolA15	PE	eBioscience	12-5698-80	1/300
MHCII	M5/114.12.2	FITC	eBioscience	11-5321-82	1/300
PD-L2	TY25	BV421	BD Biosciences	564245	1/300
Streptavidin	-	Alexa Fluor 647	Invitrogen	S21374	1/300
		BV786, BUV395, PE	BD Biosciences	563858, 564176, 554061	1/300
Sca-1	D7	FITC	eBioscience	11-5981-81	1/300
		biotin	BD Biosciences	557404	1/300
S1PR1	713412	biotin	R & D	FAB7089B	1/200

Human					
CD169	Hsn 7D2	purified	In Vitro Technologies	NB600-534	1/200
CD138	B-A38	purified	Cell Marque	138M-16	1/200
