# Supplementary material for: Memory B cells are reactivated in subcapsular proliferative foci of lymph nodes
Source: Nat Commun. 2018 Aug 22;9:3372. doi: 10.1038/s41467-018-05772-7 (PMC6105623; doi:10.1038/s41467-018-05772-7)
Supplement: Supplementary file 3 — Description of Additional Supplementary Files [file 41467_2018_5772_MOESM3_ESM.pdf]

Description of Additional Supplementary Files

File Name: Supplementary Movie 1
Description: Memory B cells localize to the subcapsular region of the B cell follicle. First ha days after HEL-OVA primary immunization. MBCs (white), CD169+ SCS macrophages (pink), SHG (blue). Second half of animation shows maximum intensity projection (3072×2048×141 µm) of same tile with spots (white) to indicate the location of MBCs. Related to Fig. 1A.

File Name: Supplementary Movie 2
Description: Naïve and MBC migration pattern. Maximum intensity projection (300×300×48 µm) of follicle from inguinal lymph node of mouse 32 days after HEL-OVA primary immunization. SWHEL MBCs (red), naïve SWHEL B cells (green) adoptively transferred 24 hours before imaging, CD169+ SCS macrophages (pink), SHG (blue), MBC SWHEL tracks (red lines), naïve B cell tracks (green lines) and CD169+ SCS macrophage surface (pink). Time stamp h:mm:ss. Related to Fig. 1B.

File Name: Supplementary Movie 3
Description: MBCs scan CD169+ SCS macrophages for antigen. Maximum intensity projection (87×72×33 µm) of follicle from inguinal lymph node of mouse 54 days after HEL-OVA immunization showing colocalization (white) of a MBC (red) with CD169+ SCS macrophages (pink). Time stamp mm:ss. Related to Fig. 1D.

File Name: Supplementary Movie 4
Description: Two distinct clusters of reactivated MBCs in the GC and SPF. First half of animation shows maximum intensity projection (504×465×31 µm) 5 days after HEL-OVA secondary immunization with SWHEL B cells (red), OT2 Tfh cells (cyan), SHG (blue), CD169+ SCS macrophages (pink). The second half of animation shows SWHEL B cell tracks (red line), CD169+ surface (pink) and GC surface (cyan) applied in Imaris. Final still frame show color codes for SWHEL B cell tracks based on migration between SPF and GC. Time stamp h:mm:ss. Related to Fig. 2, A and B.

File Name: Supplementary Movie 5
Description: Tfh cells interact with SPF cells and CD169+ SCS macrophages. Maximum intensity projection (250×250×10µm) 5 days after HEL-OVA secondary immunization showing colocalization (white) of OT2 Tfh cells (cyan) with SWHEL B cells (red) near CD169+ SCS macrophages (pink) beneath the capsule (blue). Surface contact of OT2 Tfh cells with SWHEL B cells are shown as white COLOC channel generated by Imaris. Note SWHEL B cell leaving in lymphatic flow of SCS (white triangle) in the first half of the animation. OT2 T cell track of interest is highlighted in second half of animation, and interactions of this T cell with SWHEL B cells is quantified in Fig. 2C. Time stamp h:mm:ss.

File Name: Supplementary Movie 6
Description: SPF consist of non-motile plasma cells and highly motile SPF B cells. First half of animation shows maximum intensity projection (360×360×20µm) 5 days after HEL-OVA secondary immunization. SWHEL B cells (red), Blimp-1+ (yellow, from co-expression of red and green), OT2 T cells (cyan), CD169+ SCS macrophages (pink), SHG (blue). Second half of animation shows Blimp-1+

SWHEL B cell tracks (yellow line) and Blimp -1neg SWHEL B cell tracks (red line). Time stamp hh:mm:ss. Related to Fig. 3, A and B.

File Name: Supplementary Data 1
Description: List of genes and their contributions to metagene 1.

File Name: Supplementary Data 2
Description: List of genes and their contributions to metagene 2.

File Name: Supplementary Data 3
Description: List of genes and their contributions to metagene 3.
